# *MAOA* gene hypomethylation in panic disorder—reversibility of an epigenetic risk pattern by psychotherapy

**DOI:** 10.1038/tp.2016.41

**Published:** 2016-04-05

**Authors:** C Ziegler, J Richter, M Mahr, A Gajewska, M A Schiele, A Gehrmann, B Schmidt, K-P Lesch, T Lang, S Helbig-Lang, P Pauli, T Kircher, A Reif, W Rief, A N Vossbeck-Elsebusch, V Arolt, H-U Wittchen, A O Hamm, J Deckert, K Domschke

**Affiliations:** 1 Department of Psychiatry, Psychosomatics and Psychotherapy, University of Würzburg, Würzburg, Germany; 2 Institute of Psychology, University of Greifswald, Greifswald, Germany; 3 Department of Biological Psychology, Clinical Psychology and Psychotherapy, University of Würzburg, Würzburg, Germany; 4 Division of Molecular Psychiatry, Department of Psychiatry, University of Würzburg, Würzburg, Germany; 5 Christoph-Dornier-Foundation for Clinical Psychology, Bremen, Germany; 6 Outpatient Psychotherapy Treatment Center, University of Bremen, Bremen, Germany; 7 Department of Clinical Psychology and Psychotherapy, University of Hamburg, Hamburg, Germany; 8 Department of Clinical Psychology and Psychotherapy, Technical University of Dresden, Dresden, Germany; 9 Department of Psychiatry, Marburg University, Marburg, Germany; 10 Department of Psychiatry, Psychosomatic Medicine and Psychotherapy, Goethe-University, Frankfurt, Germany; 11 Section for Clinical Psychology and Psychotherapy, University of Marburg, Marburg, Germany; 12 Institute of Psychology, University of Münster, Münster, Germany; 13 Department of Psychiatry and Psychotherapy, University of Münster, Münster, Germany

## Abstract

Epigenetic signatures such as methylation of the monoamine oxidase A (*MAOA*) gene have been found to be altered in panic disorder (PD). Hypothesizing temporal plasticity of epigenetic processes as a mechanism of successful fear extinction, the present psychotherapy-epigenetic study for we believe the first time investigated *MAOA* methylation changes during the course of exposure-based cognitive behavioral therapy (CBT) in PD. *MAOA* methylation was compared between *N*=28 female Caucasian PD patients (discovery sample) and *N*=28 age- and sex-matched healthy controls via direct sequencing of sodium bisulfite-treated DNA extracted from blood cells. *MAOA* methylation was furthermore analyzed at baseline (T0) and after a 6-week CBT (T1) in the discovery sample parallelized by a waiting time in healthy controls, as well as in an independent sample of female PD patients (*N*=20). Patients exhibited lower *MAOA* methylation than healthy controls (*P*<0.001), and baseline PD severity correlated negatively with *MAOA* methylation (*P*=0.01). In the discovery sample, *MAOA* methylation increased up to the level of healthy controls along with CBT response (number of panic attacks; T0–T1: +3.37±2.17%), while non-responders further decreased in methylation (−2.00±1.28% *P*=0.001). In the replication sample, increases in *MAOA* methylation correlated with agoraphobic symptom reduction after CBT (*P*=0.02–0.03). The present results support previous evidence for *MAOA* hypomethylation as a PD risk marker and suggest reversibility of *MAOA* hypomethylation as a potential epigenetic correlate of response to CBT. The emerging notion of epigenetic signatures as a mechanism of action of psychotherapeutic interventions may promote epigenetic patterns as biomarkers of lasting extinction effects.

## Introduction

Panic disorder (PD) is an anxiety disorder characterized by sudden, unexpected attacks of intense fear and anticipatory anxiety—often comorbid with agoraphobia—and a life-time prevalence of 1-3%.^1^ The pathomechanism of PD is genetically complex with an estimated heritability of 48%.^2^ Both cognitive behavioral therapy (CBT) and pharmacotherapy are highly effective for a large proportion of patients with anxiety disorders. However, 20–40% of all patients fail to respond sufficiently to the initial treatment along with a particularly poor quality of life, higher rates of suicidal attempts and considerable socioeconomic implications.^3^ Therefore, there is an urgent need for a better understanding of predictors and mechanisms of action of therapeutic interventions to inform expert treatment decisions towards more personalized medicine in anxiety disorders.^4^

Several biological and environmental factors have been suggested to confer PD risk and to mediate treatment success and resistance, respectively. Among those, the monoamine oxidase A (MAOA), a key enzyme in the degradation of biogenic amines such as serotonin and dopamine, is to be considered one of the prime candidates on several levels: on a pharmacological level, MAO inhibitors such as phenelzine or moclobemide are effective in the treatment of PD.^5^ On a genetic level, the more active longer alleles of a functionally relevant 30 bp variable number tandem repeat (VNTR) in the *MAOA* gene (Xp11.4–p11.3) have repeatedly been found to be associated with PD, specifically in the female subgroup of patients.^6, 7^ In a therapy-genetic study, the more active longer *MAOA* alleles predicted impaired response to CBT in patients with PD.^8^

Epigenetic processes such as methylation of the cytosine pyrimidine ring in cytosine/guanine (CpG) dinucleotides critically influence gene expression, with methylation mainly ‘silencing' DNA transcription.^9^ In the first epigenetic study in PD, we discerned hypomethylation of particularly three CpG sites in exon 1/intron 1 of the *MAOA* gene to be associated with the disorder in female patients.^10^ DNA methylation—and *MAOA* methylation in particular—has furthermore been shown to be temporally dynamic, possibly in response to environmental influences.^11^ Mirroring this dynamic nature of epigenetic processes, in female patients with PD and healthy female subjects the occurrence of negative life events was associated with relatively decreased methylation.^10^ Given the notion of temporal epigenetic plasticity to possibly constitute a key mechanism of successful fear extinction,^12, 13^ studies assessing the efficacy of therapeutic interventions to reverse epigenetic risk patterns in PD are clearly warranted.

The present study attempted to replicate our previous finding of hypomethylation in PD^10^ in an independent case–control sample. Furthermore, applying a proof-of-concept psychotherapy-epigenetic approach we for the first time investigated *MAOA* methylation changes as a potential epigenetic correlate of treatment response to a standardized 6-week CBT in adult patients with PD, as well as in an independent sample of patients with PD undergoing high-dose exposure-based CBT. Given the female-specific associations of *MAOA* variation (VNTR)^7^ and *MAOA* methylation patterns with PD,^10^ as well as the X-chromosomal location of the *MAOA* gene entailing hemizygosity in men, we restricted this analysis to all-female samples of patients with PD. Based on our previous finding of *MAOA* hypomethylation as a potential risk factor of PD,^10^ we predicted *MAOA* hypomethylation to be associated with the categorical diagnosis of PD and PD severity, respectively, as well as *MAOA* methylation to increase and thus ‘normalize' to level of controls along with response to CBT, while non-responders would show either no alteration or even a decrease of *MAOA* methylation patterns.

## Materials and methods

### Discovery sample

#### Patients

Female patients with PD (*N*=28; age (mean±s.d.): 34.57±8.51 years) with (*N*=14; 50%) or without agoraphobia were recruited at the Department of Psychiatry, the University of Würzburg, Germany, within the Collaborative Research Centre SFB-TRR-58 ‘Fear, Anxiety, Anxiety Disorders' explicitly for this study. All patients were of Caucasian background for at least two preceding generations. PD diagnosis was ascertained by experienced psychiatrists and/or clinical psychologists on the basis of a structured clinical interview (SCID-I); comorbid axis I diagnoses (except bipolar disorder, psychotic disorders, current alcohol dependence, current abuse or dependence on benzodiazepines and other psychoactive substances) were allowed if PD was the primary diagnosis (depression: *N*=12; social anxiety disorder: *N*=3; specific phobias: *N*=1). Exclusion criteria were current or previous internal or neurological somatic illnesses, any somatic medication, illegal drugs including cannabis (assessed by urine toxicology), pregnancy and excessive alcohol (>15 glasses of alcohol per week) or nicotine (>20 cigarettes per day) use. As smoking behavior has been shown to influence *MAOA* methylation,^14^ smoking status was ascertained in detail with the total number of smoked cigarettes per day during the last 4 weeks. Nine patients were classified as smokers (32%) with a total number of 4.64±7.26 (mean±s.d.) smoked cigarettes per day. Consumption of alcohol (data available for all 28 patients; *N*=14 patients reported regular alcohol consumption with 0.95±1.77 (mean±s.d.) glasses per week) and caffeine (data available for 26 patients; *N*=20 patients reported regular caffeine consumption with 10.06±8.77 (mean±s.d.) cups per week) was documented. Nineteen patients (68%) received stable psychiatric medication at baseline (selective serotonin re-uptake inhibitors, SSRIs: *N*=12; selective serotonin and norepinephrine re-uptake inhibitors, SNRIs: *N*=2; noradrenaline and selective serotonin agonist, NaSSA: *N*=4; tricyclic antidepressants, TCA: *N*=3; pregabaline: *N*=2; quetiapine: *N*=2; zopiclone: *N*=1), no patient received any other medication, and medication remained unmodified during the course of CBT (see the section ‘Treatment' below). This study was approved by the ethics committee of the University of Würzburg, Germany, and was conducted according to the ethical principles of the Helsinki Declaration. Written informed consent was obtained from all participants.

#### Treatment

PD psychotherapy in a regular outpatient clinical setting consisted of six semi-standardized sessions over ~6 weeks according to a shortened version of the exposure-based CBT manual as applied in the ‘Mechanisms of Action for CBT' (MAC) study within the BMBF network ‘Improving the Treatment of Panic Disorder'.^15^ All therapists were experienced graduate or clinical psychologists having participated in a training workshop on this manual. During the study, therapists were involved in weekly supervision to maintain therapy integrity. The first three sessions were conducted within 2 to 3 weeks each lasting ~90 min and covering psychoeducational information (for example, physiological, mental and behavioral components of anxiety, vicious circle of anxiety, vulnerability–stress model). A second three-session block within the subsequent 3–4 weeks comprised interoceptive exercises (for example, hyperventilation, straw breathing running) for all patients. Those exposure exercise sessions were conducted approximately once a week and lasted 100–240 min per session. Furthermore, these sessions were followed by intensive homework adapted to the individual's particular fears of situations. Within the last session, therapeutic gains and individual plans for continued exposure exercises, as well as relapse prevention were discussed. Medicated patients (*N*=19; see section ‘Patients' above) were only included in the study, when medication was stable for at least 2 weeks. Pharmacological treatment remained unmodified during the course of CBT. Also, patients were instructed to keep smoking behavior constant during the time course of therapy.

Given the focus on interoceptive exposure and thus the reduction of panic attacks *per se* rather than the reduction of avoidance behavior as the primary target of the current short-term, proof-of-principle treatment design (six sessions in 6 weeks), the number of panic attacks per week was assessed before (T0) and after (T1) therapy as the primary indicator of disease severity and treatment response, respectively. Patients showing a decrease in the number of experienced attacks at T1 compared with T0 (T1–T0<0) were defined as responders (*N*=11), patients not differing in the number of panic attacks or even experiencing more attacks after CBT (T1–T0⩾0) were defined as non-responders (*N*=17). Responders and non-responders did not differ regarding age, *MAOA* VNTR genotype, smoked cigarettes per day, alcohol and caffeine consumption, medication status or comorbidity with agoraphobia, depression, social anxiety disorder and specific phobias (data not shown; all *P*>0.05). The Mobility Inventory (MI)—a self-report questionnaire measuring agoraphobic avoidance in specific situations with (MI-Accompanied subscale) or without (MI-Alone subscale) company of a trusted person—was ascertained as a complementary psychometric index.^16^

#### Healthy controls

The control group consisted of 28 healthy female subjects of Caucasian descent explicitly recruited for the present study and matched to the discovery patient sample by age (age (mean±s.d.): 34.96±9.02 years; *T*=0.17, *P*=0.867) and smoking status according to the number of smoked cigarettes (eight smokers with a total number of smoked cigarettes per day of 5.59±8.82 (mean±s.d.); *T*=0.44, *P*=0.663). Absence of mental axis 1 disorders was established by experienced psychologists on the basis of a SCID (Mini International Neuropsychiatric Interview (MINI)) according to the criteria of DSM-IV. The same exclusion criteria as listed for the patient sample applied to the control sample. Healthy volunteers were evaluated at T0 and—in parallel to the course of CBT in the discovery sample—after a 6-week waiting period (T1).

### Replication sample

#### Patients

From a total sample of 154 patients included in the second multicenter (Greifswald, Münster, Würzburg, Bremen, Marburg) clinical trial of the MAC study within the BMBF network ‘Improving the Treatment of Panic Disorder',^15^ for 20 female patients (age (mean±s.d.): 33.55±11.15 years) with a primary diagnosis of PD with (*N*=14; 70%) or without agoraphobia DNA samples were available pre- and post-therapy DNA (for details see ‘DNA sampling' below). These 20 patients were thus utilized as an independent replication sample. All patients were of Caucasian descent. Diagnoses were established using a standardized computer-administered face-to-face interview (CAPI-WHO-CIDI). CIDI was administered by expert interviewers who took part in a 3-day training and a subsequent certification supervised by certified CIDI assessors of the clinical coordination center (Bremen). Inclusion criteria were: (a) a current primary DSM-IV-TR diagnosis of PD with or without agoraphobia, (b) age 18–65 years, (c) ability and availability to regularly attend treatment sessions, (d) a score ⩾4 on the Clinical Global Impression scale (CGI); comorbid axis I diagnosis (except bipolar disorder, psychotic disorders, current alcohol dependence, current abuse or dependence on benzodiazepines and other psychoactive substances) was allowed if PD with or without agoraphobia was the primary diagnosis (specific phobia: *N*=12; depression: *N*=8; social phobia: *N*=5; generalized anxiety disorder: *N*=3; obsessive-compulsive disorder: *N*=1). Exclusion criteria were current suicidal intent, borderline personality disorder, ongoing psychotherapeutic or psychopharmacological treatment for any mental disorder and physician-verified contraindications of exposure-based CBT (that is, severe cardiovascular, renal and neurological diseases). Again, smoking status was ascertained in detail with the total number of smoked cigarettes per day during the last 4 weeks. Ten patients were classified as smokers (50%), the mean number of smoked cigarettes per day in the overall sample was 6.10±7.82 (mean±s.d.). Given the exclusion criteria, none of the patients received any kind of drugs including psychiatric medication. Patients gave written informed consent after receiving a detailed description of the study program. The study was approved by the Ethics Committee of the German Psychological Society and was conducted according to the ethical principles of the Helsinki Declaration.

#### Treatment

Patients with a primary diagnosis of PD with agoraphobia received a 12-session written manualized treatment protocol focusing on *in situ* exposure to target avoidance behavior that was implemented over 6 weeks and was highly comparable to the protocol used in the first clinical trial of the MAC study^15^ with some modifications. Content, structure and doses of therapy were identical to the MAC study. Briefly, the protocol of the two different CBT variants, to which patients could be randomized, consisted of psychoeducation and an individualized behavioral analysis of the patient's symptoms and coping behavior, providing the treatment rationale for exposure and implementing interoceptive and *in situ* exposure exercises. The used protocol differed from the one of the first clinical trial only in the implementation of *in situ* exposure exercises: in both of the two possible CBT variants, the *in situ* exposure exercises were accompanied by the therapist. However, in one of the two therapy conditions, patients were additionally instructed to provoke bodily symptoms during the tasks (for example, by doing interoceptive exposure exercises). PD patients without comorbid agoraphobia received the same protocol except for *in situ* exposure exercises resulting in six therapy sessions over 3 weeks. Due to the limited sample size, we pooled all patients and analyzed the effect of therapy on *MAOA* methylation irrespective of treatment conditions.

In analogy to the discovery sample, responders (*N*=8) and non-responders (*N*=8) to CBT were defined according to the number of panic attacks at T1 compared with T0. However, given the focus on intensified exposure-based CBT mainly targeting avoidance behavior, the MI score—particularly suitable to indicate changes of pathological avoidance behavior in patients with PD and comorbid agoraphobia^16^—was chosen as the primary indicator of disease severity and treatment response, respectively.

### DNA sampling

EDTA blood was collected from all patients of the discovery sample (*N*=28) before (baseline, T0) and directly after completing the therapeutic intervention (T1). For the healthy control sample (*N*=28), EDTA blood was collected at two time points parallel to the time course of the discovery sample, that is, at T0 and after a 6-week waiting period at T1. In the replication sample (*N*=20), DNA was available for all 20 patients at T0, for 16 of them also at T1 (post therapy) and for 10 of them at T2 (follow-up at 6 months), with DNA being available at all three time points for 6 patients. DNA was isolated using the FlexiGene DNA Kit (QIAGEN, Hilden, Germany) (discovery sample, healthy control sample) or a salting out procedure (replication sample).

### *MAOA* methylation analysis

Aliquots of isolated DNA were treated with sodium bisulfite using the EpiTect 96 Bisulfite Kit (QIAGEN) according to the manufacturer's protocol for all samples in one batch and in randomized order to eliminate possible batch effects; see refs 10, 17 and 18.

An amplicon comprising *MAOA* exon 1 and parts of intron 1 (chromosome X, GRCh38.p2 Primary Assembly, NCBI Reference Sequence: NC_000023.11, 43656260–43656613) was chosen for DNA methylation analyses in analogy to previous studies on *MAOA* methylation^10, 19^ covering the CpGs most significantly associated with PD.^10^ The amplicon was PCR-amplified following a published protocol^10^ and sequenced by LGC Genomics, Berlin, Germany, on an ABI 3730XL sequencer (Life Technologies, Darmstadt, Germany).

The obtained sequences were quantitatively analyzed using the freely available Epigenetic Sequencing Methylation Software (ESME)^20^ as successfully applied previously to study DNA methylation profiles in mental disorders.^10, 17, 18, 19, 21, 22^ To account for run variability, all samples were tested in duplicate, yielding a mean individual methylation score for each CpG, as well as an individual s.d. for each duplicate. The s.d. of each duplicate was used as a first step of quality control with methylation values of duplicates with s.d.>0.1 set as missing values. In a second step, outliers (⩾3 s.d. from mean methylation of the respective CpG site) were defined as missing data. A cutoff of >20% of missing data was defined as an exclusion criterion. No participant had to be excluded from the reported analyses when applying this quality control criterion; *cf*, ref. 23.

Electropherograms were robustly readable for 13 CpG sites in patients of the discovery sample, as well as in the healthy control sample matched to the discovery sample (CpGs 1–13), and 12 CpG sites in the replication sample (CpGs 2–13), respectively. CpGs were numbered in analogy to a previous study on *MAOA* methylation in PD:^10^ CpG1=43,656,316; CpG2=43,656,327; CpG3=43,656,362; CpG4=43,656,368; CpG5=43,656,370; CpG6=43,656,383; CpG7=43,656,386; CpG8=43,656,392; CpG9=43,656,398; CpG10=43,656,427; CpG11=43,656,432; CpG12=43,656,514; CpG13=43,656,553. All non-template controls were negative. Fully methylated and non-methylated DNAs (Human Methylated & Non-methylated DNA Set, Zymo Research, Freiburg im Breisgau, Germany) were used as controls for complete bisulfite conversion.

According to published protocols with minor modifications,^10^ all patients and controls of the discovery sample were genotyped for the *MAOA* VNTR (patients: 3/3: *N*=3; 3/4: *N*=14; 3a(3.5)/4: *N*=1; 4/4: *N*=8, data missing for two patients; controls: 3/3: *N*=3; 3/4: *N*=13; 3a(3.5)/4: *N*=1; 3/5: *N*=2; 4/4: *N*=7, data missing for two controls). When grouping the sample into to low (33/34/3a4/35: *N*=18 patients; *N*=19 controls) and high expression (44/45: *N*=8 patients, *N*=7 controls) genotype groups according to previous studies,^10, 19^ patients and controls did not differ in genotype group distribution (*P*=1.000; Fisher's exact test).

### Statistical analysis

Differences in baseline methylation between PD patients of the discovery sample and matched controls were tested using mixed linear models for repeated measures; *cf*, ref. 24, with *MAOA* methylation as within factor and group (PD patients vs healthy controls) as between factor with the number of smoked cigarettes as covariate; *cf*, ref. 14, followed by univariate analysis of variance (ANOVA), again controlled for the number of smoked cigarettes (see legend of Table 1). Associations between methylation and number of panic attacks and MI score at baseline, were evaluated by Pearson's correlations.

To analyze potential dynamics in absolute methylation during therapy irrespective of treatment response, repeated measures ANOVAs with assessment time points (discovery sample: T0 vs T1; replication sample: T0 vs T1 vs T2) as within-subject variables were conducted. To evaluate possible dynamics in methylation within the healthy controls sample, repeated measures ANOVAs with two time points (T0 vs T1) as within-subject variables were calculated.

Percentage methylation change (T1–T0 in percent of T0: [T1−T0]/T0 × 100) dependent on responder status (see section ‘Treatment' above) was evaluated and controlled for baseline methylation where applicable using univariate ANOVA for average methylation, as well as for individual CpG sites. Baseline methylation differed significantly between therapy responders and non-responders at CpGs 6 (*P*=0.044) and 12 (*P*=0.026) only and were thus not included as a covariate in further analyses regarding these two CpG sites. To calculate differences in *MAOA* methylation between healthy controls and responders/non-responders at baseline (T0) and T1, respectively, multivariate ANOVAs were used. Associations between percentage methylation change (T1–T0 in percent of T0, see above) and MI score change (T1–T0) during therapy were evaluated by Pearson's correlations conducted for average methylation, as well as for individual CpG sites. For the discovery sample, *post hoc* Bonferroni correction for multiple comparisons regarding percentage methylation change at individual CpG sites (*N*=13) set the significance level to *P*⩽0.004. Given a confirmatory approach, no Bonferroni correction was applied to analyses in the replication sample. All data were normally distributed and assumption of equality of variances was met. All tests were carried out two-sided and an alpha-level of <0.05 was considered significant.

## Results

### Discovery sample

Average *MAOA* baseline methylation and methylation at the individual 13 CpGs in the discovery sample, as well as in the control sample are given in Table 1.

#### Case–control association study

Mixed linear models for repeated measures revealed that *MAOA* methylation differed significantly between PD patients and controls (*P*<0.001), with decreased average methylation in PD patients compared with healthy controls (*P*<0.001). Follow-up univariate tests revealed lower methylation in PD patients than in healthy controls at CpG sites 1–4, 6–10, 12 and 13 with *P*-values ranging from 0.049 to <0.001 (for details see Table 1). After Bonferroni correction for multiple testing, association of average *MAOA* hypomethylation, as well as hypomethylation at CpGs 3, 6–9, 12 and 13 with PD remained significant (see Table 1).

#### Baseline *MAOA* methylation and PD severity

In the patient group, at T0 a negative correlation between *MAOA* methylation and the number of panic attacks was discerned at CpG4 (*r*=−0.486, *P*=0.010). The MI-Alone subscale score at baseline correlated negatively with average baseline methylation (*r*=−0.519, *P*=0.005) and with baseline methylation at CpG3 (*r*=−0.408, *P*=0.035), CpG4 (*r*=−0.501, *P*=0.008), CpG6 (*r*=−0.491, *P*=0.009), CpG7 (*r*=−0.432, *P*=0.024) and CpG8 (*r*=−0.585, *P*=0.001). Also, MI-Accompanied scores correlated inversely with average baseline methylation (*r*=−0.470, *P*=0.013) and baseline methylation at CpG4 (*r*=−0.522, *P*=0.005), CpG6 (*r*=−0.387, *P*=0.046), CpG8 (*r*=−0.416, *P*=0.031), CpG12 (*r*=−0.405, *P*=0.036) and CpG13 (*r*=−0.450, *P*=0.018).

#### MAOA methylation change during treatment

In the overall patient group irrespective of responder/non-responder status, as well as in the control group, *MAOA* methylation did not change significantly from T0 to T1 for average methylation or at any individual CpG site (all *P*>0.05).

**Figure 1:**
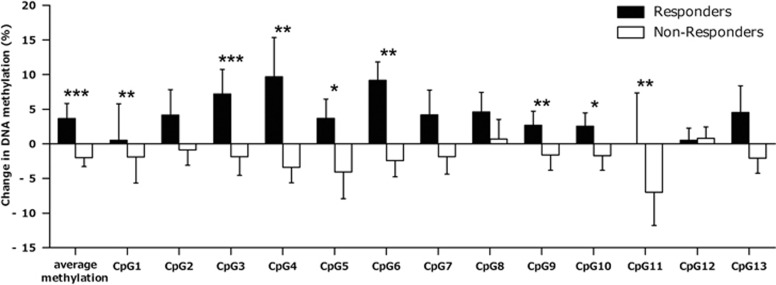
*MAOA* methylation change (% of T0; T1–T0) in responders and non-responders to CBT. Change in *MAOA* methylation (%) from baseline (T0) to post cognitive behavioral therapy (CBT; T1) in the discovery sample stratified for responders (*N*=11; black bars, mean) and non-responders (*N*=17; white bars, mean) for average methylation, as well as at single CpG sites (error bars: ±s.e.). For definition of responders/non-responders see section ‘Treatment' under ‘Patients and Methods'. *Significant at *P*<0.05; **significant at *P*⩽0.01; ***significant at *P*⩽0.001.

In the main analyses regarding percentage methylation change in the patient sample dependent on responder status according to the number of panic attacks, responders displayed an increase in average methylation after therapy (mean change±s.e., 3.37±2.17%), while non-responders decreased in average methylation (mean change±s.e., −2.00±1.28% *P*=0.001). This pattern held true for 8 out of 13 single CpG sites, with the results for average methylation and methylation at CpGs 3, 4, 6 and 11 withstanding correction for multiple testing (see Table 2 and Figure 1).

This ‘normalization' of *MAOA* methylation patterns in treatment responders was furthermore visible when comparatively assessing absolute *MAOA* methylation in responders/non-responders and healthy controls at T0 and T1, respectively: While—as expected—baseline average *MAOA* methylation differed significantly between healthy controls (mean±s.e., 0.435±0.007) and both responders (mean±s.e., 0.405±0.005; *P*=0.015) and non-responders (mean±s.e., 0.404±0.008*; P*=0.001), after 6 weeks of waiting time or CBT, respectively, average *MAOA* methylation did not differ anymore between healthy controls (mean±s.e., 0.432±0.005) and therapy responders (mean±s.e., 0.418±0.007*; P*=0.148), but remained significantly different for the contrast healthy controls vs non-responders (mean±s.e., 0.395±0.007; *P*<0.001).

In an explorative approach, an inverse correlation between changes in MI-Alone score and methylation change at CpG12 was discerned in the patient sample (*r*=−0.431, *P*=0.022).

### Replication sample

Average *MAOA* baseline methylation across all 12 CpGs readable in the replication sample (see see section ‘*MAOA* methylation analysis' under ‘Materials and Methods') was 0.39±0.06 (CpG2: mean±s.d., 0.44±0.07; CpG3: mean±s.d., 0.41±0.07; CpG4: mean±s.d., 0.35±0.09; CpG5: mean±s.d., 0.25±0.09; CpG6: mean±s.d., 0.27±0.10; CpG7: mean±s.d., 0.37±0.09; CpG8: mean±s.d., 0.25±0.08; CpG9: mean±s.d., 0.43±0.09; CpG10: mean±s.d., 0.41±0.07; CpG11: mean±s.d., 0.17±0.11; CpG12: mean±s.d., 0.90±0.09; CpG13: mean±s.d., 0.49±0.08).

#### Baseline MAOA methylation and PD severity

Prior to therapy (T0), no significant correlations between baseline *MAOA* methylation and the number of panic attacks were discerned (*N*=20; all *P*⩾0.05). However, high agoraphobic avoidance as assessed by the MI-Accompanied subscale at T0 was significantly associated with relatively decreased methylation at CpG4 (*r*=−0.46, *P*=0.047), CpG7 (*r*=−0.51, *P*=0.026), CpG8 (*r*=−0.55, *P*=0.014), CpG10 (*r*=−0.52, *P*=0.023) and CpG12 (*r*=−0.51, *P*=0.027), mirrored by a trend-wise negative correlation between avoidance and average methylation across all CpG sites (*r*=−0.43, *P*=0.064). In addition, high MI-Alone subscale scores were trend-wise associated with decreased methylation at CpG8 (*r*=−0.44, *P*=0.061).

#### MAOA methylation change during treatment

In the overall patient group irrespective of responder/non-responder status, *MAOA* methylation did not change significantly from T0 to T1 for average methylation or at any individual CpG site (*N*=16; all *P*>0.05). Accordingly, in those six patients with data available at all three time points no significant change in methylation from T0 to T2 was observed (all *P*>0.05).

Responders and non-responders defined according to the number of panic attacks did not display differences in methylation dynamics (all *P*>0.05). However, a reduction of MI-Accompanied subscale scores after therapy mostly went along with an increase in methylation as indexed by percentage increase from T0 to T1 for average methylation (*r*=−0.42), as well as for methylation at the 12 individual CpGs (*r*=−0.01–-0.57). This correlation between treatment response and increase in methylation reached statistical significance for methylation at CpG4 (*r*=−0.56, *P*=0.025), CpG7 (*r*=−0.55, *P*=0.027), CpG8 (*r*=−0.55, *P*=0.029), CpG9 (*r*=−0.55, *P*=0.028), CpG10 (*r*=−0.54, *P*=0.030) and CpG12 (*r*=−0.57, *P*=0.020), as well as trend-wise significance for CpG6 (*r*=−0.48, *P*=0.062). No significant results were observed for the MI-Alone subscale (all *P*>0.05).

## Discussion

*MAOA* hypomethylation was discerned to be associated with PD. Accordingly, PD severity was inversely correlated with *MAOA* methylation at baseline. In a psychotherapy-epigenetic approach, responders and non-responders to a 6-week standardized CBT as defined by the number of panic attacks showed differential dynamics of *MAOA* methylation during the course of treatment: response was associated with a significant increase in *MAOA* methylation up to the level of healthy controls, while non-response rather went along with a further decrease in *MAOA* methylation. Providing approximate replication of this finding, the same direction of methylation patterns and dynamics correlating with PD severity and treatment response, respectively, was identified for agoraphobic avoidance in an independent sample of unmedicated patients with PD.

The present result of *MAOA* hypomethylation to be associated with the categorical phenotype of PD, as well as with disease severity is in line with our previous observation of *MAOA* hypomethylation in PD patients,^10^ providing converging evidence for *MAOA* hypomethylation as an epigenetic PD risk pattern. Our main finding suggests dynamic changes in *MAOA* methylation as a potential correlate of response to CBT already visible after 6 weeks of therapy and thus reversibility of an epigenetic risk factor by psychotherapy. This is highly intriguing as it supports and specifies the emerging notion of epigenetically driven neuroplasticity underlying response to extinction-related psychotherapeutic interventions in anxiety disorders,^12^ already foreshadowed by Kandel's dictum ‘insofar as psychotherapy is successful in bringing about substantive changes in behavior, it does so by producing alterations in gene expression'.^25^

As in a functional *in vitro* assay decreased methylation has been shown to activate *MAOA* expression,^26^ *MAOA* hypomethylation might result in a decreased availability of monoamines in the synaptic cleft and thereby confer an increased risk for PD. This concept is supported by evidence from a positron emission tomography (PET) study using [(11)C]clorgyline showing an inverse relation between *MAOA* methylation and MAOA levels *in vivo*.^27^ Against this background and provided confirmation in future functional studies, it could be hypothesized that successful CBT reinstates functional monoamine levels by increasing *MAOA* methylation with subsequently decreased *MAOA* expression. This notion extends PET studies reporting changes in serotonin levels in patients with major depressive disorder after psychotherapy partly correlating with clinical improvement,^28^ by suggesting epigenetic processes as a possible mechanistic link.

The present study adds to the recently burgeoning body of evidence for epigenetic mechanisms possibly constituting dynamic biological correlates of therapeutic interventions. In this respect, our results suggesting reversibility of *MAOA* hypomethylation by CBT complement a previous study by Roberts *et al.*^29^ reporting increases in *5-HTT* gene methylation to correlate with remission status after CBT in a sample of children with mixed primary anxiety disorder diagnoses. To the best of our knowledge, so far only four other studies have investigated the dynamics of epigenetic processes as a potential correlate of treatment response in mental disorders or related animal-model phenotypes: In rats with a depression-like phenotype (flinders sensitive line), *P11* gene hypermethylation was found to be reversible by antidepressant treatment with escitalopram.^30^ In patients with major depression, Lopez *et al.*^31^ discerned a significant decrease in histone H3 lysine 27 trimethylation (H3K27me3) levels at promoter-IV of the *BDNF* gene along with response to citalopram after 8 weeks of treatment. In patients with borderline personality disorder, initially increased *BDNF* gene methylation decreased during a 4-week course of intensive dialectical behavior therapy along with therapy response.^32^ Finally, Yehuda *et al.*^33^ reported *FKBP5* methylation to decrease in association with responder status after a 12-week psychotherapy in combat veterans with post-traumatic stress disorder. Given that presently no changes in *MAOA* methylation were discerned in age- and sex-matched healthy probands when applying a control study design paralleling the time course of psychotherapy in the discovery patient sample, it can be assumed that the changes in *MAOA* methylation discerned in patients are due to psychotherapy effects. The present observation of non-response to CBT to be accompanied by a further decrease in *MAOA* methylation underlines some reports of temporary symptom exacerbation during exposure therapy, for example, in a minority of patients with post-traumatic stress disorder particularly when only considering the very early-treatment stages, for example refs. 34 and 35, and suggests *MAOA* methylation dynamics as a potential epigenetic correlate.

Despite several strengths such as high clinical and demographic homogeneity of the present patient sample, strict inclusion/exclusion criteria, recruitment of a matched healthy control sample, consideration of several potential confounders of epigenetic processes, survival of our main results after conservative Bonferroni correction, as well as the attempt of a replication in an independent sample, the present findings have to be interpreted in the light of some limitations and remarks: owed to the proof-of-concept design, strict inclusion/exclusion criteria and inclusion of female patients only due to the X-chromosomal location of the *MAOA* gene, sample sizes—particularly of the replication sample, which was not explicitly recruited for this purpose—are relatively small warranting further replication in larger samples including male patients, as well as applying a follow-up design. An *a priori* calculation of the required total sample size was hindered by the fact that the only published study regarding the relationship between changes in methylation patterns and psychotherapy response in anxiety disorders suggesting medium effect sizes was not fully comparable to the present study given a different study population (children), heterogeneity of anxiety diagnoses (generalized anxiety disorder: *N*=56, specific phobia: *N*=20, separation anxiety disorder: *N*=16, social anxiety disorder: *N*=14, obsessive-compulsive disorder: *N*=9, PD/agoraphobia: *N*=1), a different response criterion and a different gene of interest (*5-HTT*; *SERT*).^29^ However, the presently observed medium to large effect sizes (*cf*, Tables 1 and 2) suggest sufficient power to detect these effects. Second, focus and intensity of CBT in the discovery and the replication sample were not fully comparable (discovery sample: 6 sessions in 6 weeks, focus on interoceptive exposure; replication sample: 12 sessions in 6 weeks, focus on *in situ* exposure). Also, the clinical composition of the samples regarding comorbidity with agoraphobia was different (discovery sample: 50% replication sample: 70%). Against this background, it is remarkable that the observed methylation changes as a potential correlate of treatment response applied to different response criteria, that is, panic attacks *per se* (discovery sample) and avoidance behavior as measured by the MI score (replication sample), potentially reflecting differences in treatment focus and intensity and/or comorbidity rates between the two samples. Thus, replication of the present finding in the current design is to be considered approximate and warrants further evaluation in large independent samples exhibiting a highly parallelized clinical design. Medication with antidepressants might influence methylation status on the one hand, for example ref. 30, and has been shown to potentially aggravate PD symptoms during uptitration of the drug on the other hand.^36^ However, as in the discovery sample (i) all patients receiving psychotropic medication were stable on a clinically sufficient dose of the respective medication for at least 2 weeks before inclusion into the study, (ii) medication was not changed during CBT, (iii) baseline *MAOA* methylation differed between medicated and non-medicated patients at one single CpG only and most importantly did not statistically impact methylation change during therapy, and (iv) patients in the replication sample were entirely medication-free, this was probably not a major confounding factor in the present study. Smoking as another possible confounder of the present results—given its known role as a modulator of *MAOA* methylation^14^ and as a potential causal factor/facilitator in the development of PD^37^—has been controlled for on several levels: (i) smoking status was ascertained in detail in both the discovery and the replication sample, (ii) patients were instructed to keep smoking behavior constant during the time course of therapy, (iii) the control group was matched to the discovery patient sample according to smoking status, and (iv) all tests regarding baseline methylation were controlled for smoking behavior. Technically, DNA methylation was measured in blood samples entailing cell-composition effects as a possible confounder. Finally, investigation of epigenetic patterns in peripheral biomaterial such as blood does not allow for direct conclusions regarding methylation correlates in brain tissue.^10^ However, a recent PET study reporting peripheral *MAOA* methylation in leukocytes to inversely correlate with brain MAOA levels suggests *MAOA* methylation in blood as a viable sensor for central processes.^27^ In general, the role of *MAOA* methylation in the pathogenesis and/or treatment response mediation in PD needs to be confirmed by epigenome-wide association studies.

In summary, the present psychotherapy-epigenetic study adds to previous evidence for *MAOA* hypomethylation as a risk marker of PD and for the first time suggests *MAOA* methylation changes as a potential epigenetic correlate of treatment response to CBT in PD patients. Given robust replication of the present results, *MAOA* methylation pattern as an accessible biomarker of PD risk may aid in developing resilience-increasing preventive measures for epigenetically defined high-risk groups. In addition, the emerging notion of epigenetic signatures as a core mechanism of action of response to psychotherapeutic interventions is hoped to contribute to the development of a more personalized and thus more effective treatment of PD. For instance, along the lines of an individualized treatment augmentation approach, MAO inhibitors could be probed as an adjunct to psychotherapy in PD patients displaying *MAOA* hypomethylation. On a more general note, the present findings might contribute to the promotion of psychotherapeutic or pharmacological options inducing epigenetics changes for lasting extinction effects.

## Figures and Tables

**Table 1:** *MAOA* methylation in the discovery sample of patients with panic disorder and healthy controls at baseline (T0)

	***Patients (*N=*28)*** ***M (s.e.)***	***Controls (*N=*28)*** ***M (s.e.)***	**P-*value***	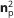
Average methylation	0.404 (0.005)	0.435 (0.007)	<0.001***	0.282
CpG1	0.341 (0.008)	0.369 (0.011)	0.033*	0.121
CpG2	0.326 (0.006)	0.358 (0.010)	0.019*	0.138
CpG3	0.343 (0.007)	0.387 (0.011)	<0.001***	0.275
CpG4	0.395 (0.008)	0.420 (0.007)	0.049*	0.108
CpG5	0.268 (0.008)	0.281 (0.011)	0.169	0.065
CpG6	0.321 (0.008)	0.364 (0.008)	<0.001***	0.250
CpG7	0.414 (0.005)	0.450 (0.006)	<0.001***	0.314
CpG8	0.288 (0.007)	0.330 (0.010)	0.004**	0.190
CpG9	0.442 (0.006)	0.478 (0.006)	0.001***	0.238
CpG10	0.461 (0.005)	0.485 (0.007)	0.013*	0.152
CpG11	0.317 (0.013)	0.293 (0.010)	0.233	0.053
CpG12	0.870 (0.009)	0.910 (0.008)	<0.001***	0.374
CpG13	0.473 (0.010)	0.553 (0.015)	<0.001***	0.369

Abbreviations: ANOVA, analysis of variance; VNTR, variable number tandem repeat.

Age, *MAOA* VNTR genotype, comorbidity with agoraphobia or depression and medication did not influence average *MAOA* methylation at baseline (T0; all *P*>0.05) in PD patients. However, the number of smoked cigarettes per day correlated inversely with average *MAOA* methylation (*r*=−0.379, *P*=0.047). In the control sample, age and *MAOA* VNTR genotype did not influence average *MAOA* methylation (all *P*>0.05). Again, the number of smoked cigarettes per day correlated inversely with baseline *MAOA* methylation at CpGs 3, 12 and 13 (*r*=−0.540 to −0.400, all *P*<0.05). Thus, all analyses at T0 were corrected for the number of smoked cigarettes per day. *P*-value (average methylation) from mixed linear model with number of smoked cigarettes as covariate; *P*-values (single CpG sites) from univariate ANOVA corrected for number of smoked cigarettes. *Significant at *P*<0.05; **significant at *P*<0.01; ***significant at *P*<0.001; bold=significant after Bonferroni correction for multiple testing. 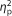, partial eta squared is reported as an estimate of effect size.

**Table 2:** *MAOA* methylation change (% of T0; T1–T0) for responders and non-responders to CBT in the discovery sample of patients with panic disorder

	***Responders (*N=*11)a*** ***M (s.e.) (%)***	***Non-responders (*N=*17)a*** ***M (s.e.) (%)***	**P-*value***	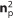
Average methylation	3.37 (2.17)	−2.00 (1.28)	0.001***	0.415
CpG1	0.51 (5.28)	−1.90 (3.75)	0.005**	0.341
CpG2	4.15 (3.69)	−0.87 (2.23)	0.087	0.178
CpG3	7.22 (3.53)	−1.85 (2.70)	0.001***	0.446
CpG4	9.68 (5.67)	−3.40 (2.23)	0.003**	0.365
CpG5	3.70 (2.77)	−4.08 (3.82)	0.040*	0.228
CpG6	9.18 (2.65)	−2.42 (2.33)	0.003**	0.286
CpG7	4.19 (3.58)	−1.85 (2.54)	0.098	0.170
CpG8	4.60 (2.84)	0.69 (2.84)	0.085	0.179
CpG9	2.68 (2.04)	−1.62 (2.19)	0.009**	0.313
CpG10	2.57 (1.91)	−1.70 (2.12)	0.027*	0.251
CpG11	0.01 (7.34)	−6.99 (4.79)	0.002**	0.390
CpG12	0.50 (1.77)	0.82 (1.62)	0.898	0.001
CpG13	4.54 (3.86)	−2.06 (2.21)	0.276	0.098

Abbreviations: ANOVA, analysis of variance; T0, baseline; T1, post cognitive behavioral therapy; VNTR, variable number tandem repeat.

No influence of age, *MAOA* VNTR genotype group, comorbidity with agoraphobia or depression, medication or smoking behavior were detected on percentage average *MAOA* methylation change (T1–T0). However, methylation at T0 influenced average *MAOA* methylation change (*r*=−0.545, *P*=0.003), necessitating control for methylation at T0 for analyses regarding methylation dynamics. *Significant at *P*<0.05; **significant at *P*⩽0.01; ***significant at *P*⩽0.001; bold=significant after Bonferroni correction for multiple testing. 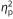, partial eta squared is reported as an estimate of effect size.

a Definition of responders/non-responders, see section ‘Treatment' under ‘Patients and Methods' *P-*values from univariate ANOVA controlled for baseline methylation of the respective CpG site.
